# Mimicking effects of auditory verbal hallucinations on language production at the level of words, sentences and stories

**DOI:** 10.3389/fpsyg.2022.1017865

**Published:** 2022-11-16

**Authors:** Stefan Heim, Stella Polyak, Katja Hußmann

**Affiliations:** ^1^Institute of Neuroscience and Medicine (INM-1), Forschungszentrum Jülich GmbH, Jülich, Germany; ^2^Department of Psychiatry, Psychotherapy and Psychosomatics, Medical Faculty, RWTH Aachen University, Aachen, Germany; ^3^Department of Neurology, Medical Faculty, RWTH Aachen University, Aachen, Germany

**Keywords:** schizophrenia, simulation, auditory interference, language production, disorganised speech, neurotypical adult volunteers

## Abstract

Schizophrenia is characterised foremost by hallucinations, delusions and disorganised speech. Deficits in the internal speech monitor may contribute to the development of auditory-verbal hallucinations. This study investigates potential effects in the opposite direction: could the presence of auditory-verbal hallucinations have an effect on speech production? To this end, a recent mimicking/simulation approach was adopted for 40 healthy participants who perceived either white noise or hallucination-like speech recordings during different language production tasks with increasing demands: picture naming, verbal fluency with and without category switch, sentence production, and discourse. In line with reports about real schizophrenia cases in the literature, mimicking auditory-verbal hallucinations affected verbal fluency (switch condition) and sentence production (duration) in a different way than mere noise. These effects were not correlated, suggesting that hallucinations may even affect different levels of linguistic complexity in different ways. Anyway, in both cases (mimicked), auditory hallucination appear to contribute to the emergence of disordered speech. The mimicking/simulation paradigm may in future help to identify and disentangle the various factors contributing to disorganised speech in schizophrenia. They may also support the development and implementation of new protocols, e.g., in speech and language therapy in persons with schizophrenia in order to improve their communication skills despite the presence of auditory-verbal hallucinations.

## Introduction

Disorganised speech is one of the three most prominent symptoms of schizophrenia. According to the DSM-5, two symptoms out of the following must be present for at least one month (Tandon et al., 2013): delusions, hallucinations, and disorganised speech. Language use in schizophrenia shows systematic abnormalities at various linguistic levels from the single word to the connected complex utterance, suggesting its role even as a potential biomarker for schizophrenia (e.g., Covington et al., 2005; de Boer et al., 2020a,b; Voppel et al., 2021). Problems in lexical retrieval (word finding) are reflected in lower scores in verbal fluency tasks (e.g., Juhasz et al., 2012; Creyaufmüller et al., 2020) and in neologisms (involuntary creations of novel or nonsense words) and semantic paraphasias (selection of the wrong word from the same semantic domain, e.g., “cat” for “dog”). Also, the learning of new words is impaired (Tunkel et al., 2014). At the sentence/text level, narrative and pragmatic problems are found (tangentiality, derailment): persons with schizophrenia (PwS) lose track of their topic, follow chains of loose associations, repeat utterances or ignore rules of turn-taking etc. (e.g., Covington et al., 2005; Marini et al., 2008; Colle et al., 2013; Bambini et al., 2016). These problems in language productions go along with disturbances in understanding irony, humour, or metaphors (e.g., Kuperberg, 2010a; Perlini et al., 2018; Tavano et al., 2008). Syntactic problems also occur in production (incomplete sentences, simpler syntactic structures: Stassen et al., 1995; Perlini et al., 2012; Zimmerer et al., 2017) and comprehension (difficulties with complex syntactic structures; e.g., Condray et al., 2002; Kuperberg, 2010a; Perlini et al., 2012; Delvecchio et al., 2019).

Despite the relevance of linguistic deviations in schizophrenia, hallucinations are commonly regarded as a far more prototypical sign of the disease. In fact, 70–80% of PwS develop acoustic, visual and/or tactile hallucinations (Gaser et al., 2004), often subsequent to other prodromal Axis-I diagnoses (DSM-IV terminology; *cf.* the seminal work of the Klosterkötter group, e.g., Salokangas et al., 2012). The acoustic hallucinations are of particular relevance as they are often perceived as imperative, commenting, or dialogic voices. Conde et al. (2016) give an overview of the four major theoretical accounts and their behavioural and neuroscientific evidence: memory-based; reality-monitoring; auditory vivid-imagery; and verbal self-monitoring. These accounts draw upon the well-documented alterations in three aspects of voice processing: semantic content in the voice, identity (self vs. other) transported *via* the voice, and affective information contained in the voice. Each of these aspects is associated with functional alterations in different parts of the brain. Whereas the first two accounts may describe primary or even causal mechanisms, the latter might add the predominantly negative valence of the contents of auditory verbal hallucinations. For instance, de Boer et al. (2019) showed stronger effects of verbal than of non-verbal auditory hallucinations. In addition, Rossell and Boundy (2005) provided evidence for the interaction of alterations in both the semantic and the affective domain.

The identity account and the semantic account have in common the notion of a deficit in top-down processing. Auditory verbal hallucinations might be caused by a dysfunction of the speech output monitoring system, i.e., the monitoring of inner speech (Hubl et al., 2004; Vercammen et al., 2008), causing PwS to perceive their own thoughts as external voices (Allen et al., 2007; Cho and Wu, 2014; Pinheiro et al., 2019; Whitford, 2019). Hugdahl and Sommer (2018) suggested that hallucinations may lead to gating effects and/or extended refractory periods in auditory cortex neurons, causing involuntary shifts of attention. Hugdahl (2009) referred to this effect as a failure of top-down control to sensory processes. In a dichotic listening study, Hugdahl et al. (2013) demonstrated that this failure could selectively occur for the one or other ear, depending on which channel had to be attended. Along the same lines, Løberg et al. (2015) related the emergence of auditory-verbal hallucinations to systematic attention deficits (see also Waters et al., 2012). Interestingly, the connection of speaking and auditory hallucinations has in fact a neurobiological basis: the connectivity strength of speech and auditory brain areas is associated with the presence or severity of auditory-verbal hallucinations (Li et al., 2017; Zhang et al., 2018).

It is yet an open issue whether this relationship between hallucinations and disorganised speech works only one-way, i.e., whether a disorder in the speech monitor influences/causes hallucinations, or rather both ways: does the presence of auditory hallucinations have a potential disorganising impact on the speech output system? For instance, the monitoring deficit hypothesis may not fully account for the fact that PwS with auditory-verbal hallucinations have lower scores in semantic verbal fluency than those without (Siddi et al., 2017).

An empirical test of the question whether the presence of auditory-verbal hallucinations affects language production is difficult in real PwS: people with acute psychosis only show a limited tolerance to extensive neuropsychological and neurolinguistics testing. Later, on medication, their hallucinations may decline, making it difficult to trace any residual connections to language. Moreover, it might be difficult singling out the individual effects of hallucinations from all the other symptoms in order to assess their selective influence on speaking. One potential solution to this problem is the use of mimicking/simulation paradigms in healthy volunteers in which states are induced that bear a relevant resemblance to those of PwS in their acute phase of a disease (for a review on schizophrenia *cf.* Ando et al., 2011; for other mimicking studies *cf.* the review by Heim, 2013). Such mimicking/simulation accounts have demonstrated their usefulness in modelling the behavioural and neural mechanisms underlying developmental dyslexia (Tholen et al., 2011; Heim et al., 2014) and aphasia (Meffert et al., 2011; Grande et al., 2012). Even though this approach has limitations, in particular that the complex constellation of a disorder is at best approximated but never fully realised, there are several potential advantages: larger, well-described samples with clearly defined inclusion criteria can be recruited systematically; the participants can endure longer testing sessions at lower stress levels; individual factors can be isolated and manipulated individually, allowing the systematic experimental control with increasing complexity; and replication studies are easier feasible. A mimicking/simulation account cannot provide a final truth, but it allows pursuing or generating hypotheses to be tested in real PwS who were spared from participating in all the trials and errors in the piloting phases.

Importantly, in the field of schizophrenia, several such studies simulating/mimicking visual (Rastelli et al., 2022) and predominantly auditory hallucinations (Brown, 2010; Ando et al., 2011; Evans et al., 2015; Kidd et al., 2015; Kepler et al., 2016; Skoy et al., 2016; Silva et al., 2017; Kim and Wojnar, 2019; Riches et al., 2018, 2019; Duprey et al., 2021) were published in the past years. The major goal was to raise awareness and empathy for the particular situation of PwS who suffer from such hallucinations. Simulations were applied to students of different health professions, and their self-reports on their experiences and also effects on behavioural performance measures were assessed. For instance, in the study of Silva et al. (2017), auditory-verbal hallucinations were mimicked based on transcripts of reports from schizophrenia patients. Two actors recorded audio tracks which were presented to healthy volunteers. Real human voices instead of computer-generated stimuli were used to increase the authenticity. Kim and Wojnar (2019) obtained data for disturbance in the domains of attention (alertness, concentration, train of thoughts) and memory (memorise and recall), for increase of unpleasant emotions and somatic changes (e.g., racing heart, shallow breath, higher blood pressure and heart rate), and odd behaviour (e.g., fidgeting, nail biting, fiddling with hair and clothes, repeated time checking, inappropriate laughing, swearing and self-talking). In fact, for 90% of their participants, there were changes in their degree of functioning and performance. The authors thus concluded that these mimicked hallucinations “closely resembled the voice hearers’ actual experience” (Abstract, p: 240). “Conceivably students may have reacted to the voice-hearing experience in a manner similar to patients’ experiences of post-psychosis emotional disturbance (Birchwood, 2003). […] The distinctive difference between patients and students was the use of self-reminders and reassurances by faculty that VHS would end soon and the option of stopping the voices was available at any time.” (p. 245). Diederen et al. (2012) investigated the neural similarity of mimicked hallucinations, actual hearing under auditory stimulation, and imagination of voices using positron emission tomography. The activation patterns for hallucinations and auditory stimulation were very similar to each other and quite distinct from imagination.

Whereas the goal of these simulation studies was to increase empathy in unaffected people (e.g., in the education of nurses or pharmacists) with the situation of PwS, Kim and Wojnar (2019) also reported a wide range of behavioural effects. Thus, the procedure might likewise be used to investigate the selective effects of the presence of commenting, imperative or dialogue-like voices on verbal behaviour, i.e., the speech-output system in healthy volunteers. Consequently, the present study used this approach, which in consequence may contribute to generating novel hypotheses about the potential loci in the cognitive system at which real hallucinations in real PwS might act. The paradigm is thus an extension of the well-established picture-word interference task in which the presence of distractor stimuli may alter the speed or accuracy of the intended utterance (e.g., Klaus et al., 2017 or Creyaufmüller et al., 2020 for application in both healthy persons and PwS). It is feasible and valid on the grounds that PwS with auditory-verbal hallucinations fail to distinguish external from internal voices, at least in those stages of the disease in which the PwS have no sense of distance to the hallucinations. Moreover, if it mimics disorders in the speech output, these effects will go beyond the standard Lombard effect (Lombard, 1911) that speakers raise their voice when in noisy environments, because here noise serves as the baseline. In the present study, all speech output levels from single-word retrieval (verbal fluency, picture naming) to sentence production and discourse were addressed, thus offering a systematic and comprehensive investigation of the effect.

## Materials and methods

The study was approved by the local ethics committee at the Medical Faculty, RWTH Aachen University (EK080/19).

### Sample

Forty healthy adults (aged 20–30 years; 19 women) participated in the study. We recruited volunteers in this age range (mostly amongst the students at RWTH Aachen University, plus *via* some personal contacts). The age range was chosen since the majority of people with schizophrenia encounter their first episode at this age (Buchanan and Carpenter, 2005). The only other inclusion criterion was a self-report of native or native-like proficiency of German. Exclusion criteria were a personal or family history of psychiatric disorders or mental disability, acquired language disorders at the time of testing (e.g., aphasia), and auditory and/or visual sensory deficits. Finally, immediately before the start of the experiment, all participants were assessed for signs of depression with the short version of the Beck Depression Inventory (BDI; Kühner et al., 2007; *cf.* Schmitt et al., 2006) since they were going to be confronted with potentially aversive auditory materials (see Appendix A) which might induce negative affect. Volunteers scoring high on one or more of these items (i.e., scores >1) would have been excluded. No volunteer had to be excluded in this screening. We did no further cognitive assessment of the volunteers in order not to expand the total duration of the study. The rationale was that, if any effects were found, a follow-up study focussing only on these effects could involve more targeted cognitive testing.

### Mimicking auditory-verbal hallucinations

In order to create materials for mimicking auditory-verbal hallucinations, we followed the procedures by Silva et al. (2017). First, transcripts were created based on an extensive literature review of the contents of real auditory-verbal hallucinations and on the expertise of author SH, professor at the Department of Psychiatry. In addition, a colleague psychiatrist from RWTH Aachen University Hospital was interviewed as to any further contents and way of “speaking” of the voices, as reported by PwS. This helped create 16 min of commenting, imperative, or dialogical voices. The type of voice changed in rhythms of 1 min in a way unpredictable to the participants, in correspondence with reports from clinical cases. Contents included suicide, self-hatred and isolation/loneliness but also offhand inclusions of every-day objects or episodes (see Table 2 in the Supplementary Material). These transcripts were then spoken by one male and one female speaker and recorded in the Audio-Visual Media Centre (AVMZ) at RWTH Aachen University Hospital. In order to render the simulation more naturalistic, the recordings were edited with *Avid Pro Tools* (Collins, 2004) to achieve fluctuations in frequency and volume, thus creating the impression of more than two voices. The AVMZ also created 16 min of white noise stimuli with comparable acoustic properties. The audio files were presented *via* headphones while the participants performed their tasks, thus ensuring that the stimuli were not obliterated by external noise.

### Tasks and materials

The study investigates language production at the level of words, sentences, and stories. Accordingly, the following tasks were used: (1) verbal fluency, (2) verbal fluency with category switch, (3) picture naming, (4) sentence production (subject-predicate-object format), (5) discourse. Each task was composed in two parallel versions such that the influence of mimicked auditory-verbal hallucinations during each task could be compared to a baseline condition with white-noise as background audio signal. The simulated auditory-verbal hallucinations were not temporally aligned to the different tasks but just continued without pause, as reported in clinical cases. Thus, there was no systematic balancing of the contents of the simulated hallucinations and the tasks. If the presence of the simulated hallucinations has any effect on speaking performance, a systematic investigation of potential influences of the exact content can be performed in a subsequent study.

#### Verbal fluency

Verbal fluency is slightly more challenging than picture naming since there is no concrete object to be named but exemplars must be generated from memory. In this study, the standard German verbal fluency test (Regensburger Wort-Flüssigkeits-Test, RWT; Aschenbrenner et al., 2001) was used. It consists of two versions, the simple fluency condition (both semantic, e.g., “surnames” and phonological, e.g., “words starting with the phoneme S”) and the switch condition (alternating between two semantic categories “types of sports/fruits” or two phonemes “G”/“R”). In each condition, participants are asked to generate as many exemplars to the criterion as they can retrieve in 2 mins. The switch conditions pose additional requirements on executive functions as compared to the simple conditions. There are normative values available for different age groups, allowing the transformation of raw scores into percentiles of the respective age group. Consequently, the different versions of simple fluency can be compared among each other, and likewise the various switch conditions. The percentile values per condition/test were used for the analysis.

#### Picture naming

Picture naming was the most simple task in the study: black-and-white drawings of every-day objects from the Snodgrass & Vanderwart set (*cf.* Szekely et al., 2005) were used to create two parallel sets of pictures of every-day objects to be named. Each version contained 48 pictures controlled for number of syllables (16 per 1/2/3 syllables) and lexical frequency (dlexDB database, 2008–2012).^1^ A two-samples t-test confirmed that the distribution of frequencies was comparable in the two sets (1629.3/mio vs. 1795.9/mio; *t*[94] = −0.223; *p* = 0.824). The order of items with 1/2/3 syllables was also kept parallel between the two sets, thus creating maximal comparability of the items for naming while hearing auditory-verbal materials or white noise. Each picture was presented for 3 s. The following parameters were assessed: accuracy of the picture name, speech latency, and duration of the word. Also, the occurrence of semantic paraphasias was analysed.

#### Canonical SPO sentences

For the production of language beyond the single-word level, stimuli were also taken from the set of black-and-white drawings (Szekely et al., 2005). In contrast to simple picture naming, these stimuli showed actions performed by one agent with one object. Two parallel versions of 24 pictures each were created. Participants had to utter sentences in the canonical Subject-Predicat-Object (SPO) word order (e.g., “The man is throwing the ball”). The following parameters were recorded: accuracy of the sentence (syntactically complete and semantically correct), speech onset latency, and duration of the utterance.

#### Stories

For discourse, the use of published materials is a standard procedure (e.g., DeLisi, 2001; Perlini et al., 2012). In the present study, materials for learning Dutch as a foreign language (Schneider-Broekmans, 2015) were used for eliciting story generation. They each contain of eight black-and-white drawings of every-day situations (e.g., in a café) which have to be integrated into one story within 1 minute. Two sets of four stories each were created. From the audio recordings of connected speech during discourse, lexical information units (LIUs) and thematic information units (TIUs) were extracted and analysed (e.g., Marini et al., 2008). LIUs are all content words (e.g., nouns, verbs, adjectives) and function words (e.g., articles, conjunctions, prepositions) that were phonologically, syntactically and pragmatically correct/appropriate. The number of all such words was divided by the total number of uttered words (containing also, e.g., paraphasias, repetitions, interjections, jargon etc.) and converted into a percent score. Similarly, for each story, the number of uttered thematic topics was assessed and divided by the number of expected topics. This score, again, was converted into a percent value.

#### Parameters

Psycholinguistic studies of language production usually make use of the following parameters: *Accuracy* of the response (i.e., success of retrieval; mostly used in patient studies and/or cognitive modelling of patients’ performance: e.g., Dell (1986) model. For a recent paper, *cf.* Middleton et al., 2022); *Latency* of the response (i.e., reaction time, response speed; mostly used in studies with neurotypical participants and/or cognitive modelling of their responses: e.g., Willem Levelt’s (Levelt et al., 1999) model. For a recent paper, *cf.* Mascelloni et al., 2021); and *Duration* of the response (for longer utterances beyond single words; e.g., (Sarkis and Montag, 2021). Moreover, in verbal fluency tasks, a fixed time interval (usually 1 min or 2 min) is given and the number of words correctly produced in this interval counted. In the present study with its exploratory character, we sought to discover all possible effects. Therefore, accuracy was measured where possible (i.e., correct lexical retrieval of words and correct production of SPO sentences). Likewise, response latencies and response durations where measured whenever a stimulus was presented to which one particular response (target word, SPO sentence) was expected. This was not possible for verbal fluency, for which the number of words per time unit was assessed accordingly and then transformed into the corresponding normative value (percentile). Likewise, for discourse, speech latencies and duration would not be informative because of the complexity beyond a single word or short sentence. Therefore, the lexical richness of the utterances was assessed with the TIUs and LIUs.

### Design and procedure

All participants performed all tasks under all experimental conditions. The tasks were administered in a fixed order: verbal fluency (simple, switch), picture naming, sentence production, discourse. Verbal fluency was tested before picture naming in order not to prime any lexical items that might have occurred previously as target pictures. The presentation of the stimulus pictures was controlled by a PC running on Microsoft Windows. One run of all tasks had a duration of 16 minutes, totaling 32 minutes for the two runs with mimicked auditory-verbal hallucinations in the one, and white noise in the other. In order to prevent any order effects with respect to the type of auditory background sounds (mimicking hallucinations), the order was counter-balanced across participants: one half of the participants received the auditory-verbal hallucinations in the first and the white noise in the second run while the order was reversed in the other half of participants.

### Data analysis

The statistical analysis was performed with IBM SPSS 24.^2^ For each participant, task, parameter and condition, the data were aggregated. For each task and parameter, a 2×2 ANOVA was performed with “*Condition*” (hallucination/noise) as within-subject factor and “*Order*” (order of the two auditory backgrounds) as between-subject factor in the sense of a covariate to partial out any remaining order effects that might not have been covered by counter-balancing the order of presentation. The significance threshold of *p* < 0.05 was Bonferroni-corrected for multiple comparisons separately for each task that came in different varieties (RWT simple/switch) or with sets of dependent variables affording multiple tests (total accuracy/latency/duration for picture naming and for sentence production; LIU/TIU for discourse). In these analyses, the main effect for *Condition* was the one effect of interest. For the sake of completeness, the main effects of *Order* and interactions are also reported. These latter, however, have no immediate significance for the argument itself, as the present study did not investigate the potential habituation to mimicked auditory verbal hallucinations (*cf.* e.g., Kim and Wojnar, 2019). The *Order* effects are, however, potentially relevant for follow-up studies in which a more fine-grained manipulation (e.g., investigation of the effects of the different types of voices) might interact with the order of presentation.

In the light of the emerging results (see below), two additional analyses were run. First, as there was a significant finding in the fluency-switch condition but not in the fluency-simple condition, suggesting a systematic difference of the simulation in two tasks, we ran a confirmatory 2 × 2 × 2 ANOVA with “Task” (fluency-simple/fluency-switch) and “Condition” (hallucination/noise) as between-subject factors and “Order” as between-subject factor, testing for an interaction of the two within-subject factors. Moreover, as significant effects of *Condition* occurred for both fluency-switch and for sentence production (duration), we tested for potential relationships between the two by a set of Pearson correlations (due to the exploratory nature of this analysis, no value of p correction was applied).

## Results

**Figure 1:**
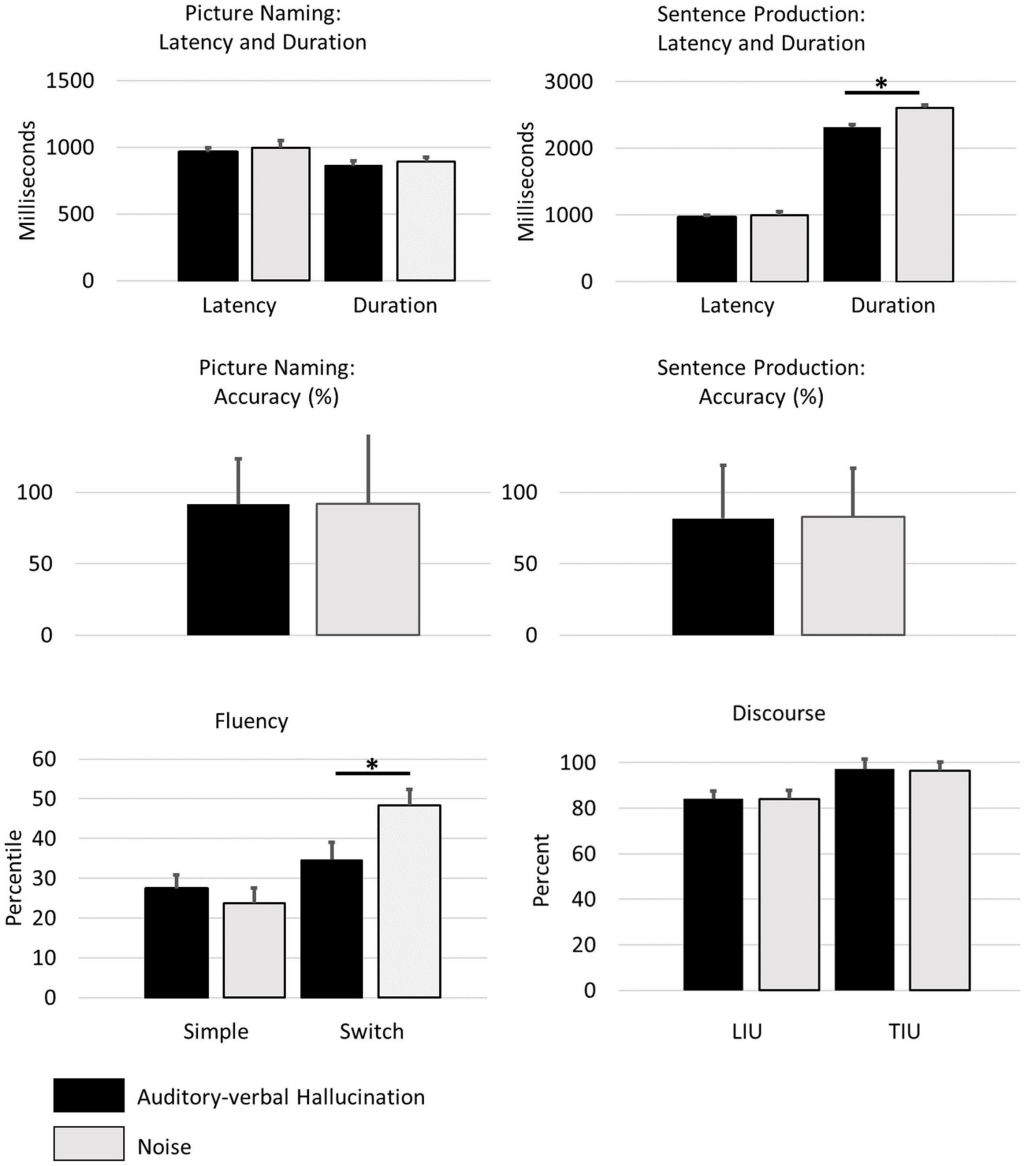
Average performance in the different language production tasks under auditory-verbal hallucinations (black) and noise (grey). Error bars indicate the standard error of the mean. * *p* < 0.05 corrected for multiple comparisons within the task.

The average performance on each of the parameters in the different tasks and conditions is displayed in Figure 1. The results of the 2 × 2 ANOVAs with factors *Condition* and *Order* are summarised in Table 1.

**Table 1:** Results of the 2 × 2 ANOVAs for effects of *Condition* and *Order* and their interaction.

Task	Parameter	Main effect of Condition	Main Effect of Order	Interaction Condition x Order
Verbal fluency, simple	Percentile (items per time unit)	*F*[1;38] = 0.511 *p* = 0.479	*F*[1;38] = 0.110 *p* = 0.742	*F*[1;38] = 86.496 *p* < 0.001
Verbal fluency, switch	Percentile (items per time unit)	*F*[1;38] = 7.042 *p* = 0.012	*F*[1;38] = 3.370 *p* = 0.074	*F*[1;38] = 0.078 *p* = 0.782
Picture naming	Accuracy	*F*[1;38] = 0.087 *p* = 0.770	*F*[1;38] = 10,892.569 *p* < 0.001	*F*[1;38] = 3.569 *p* = 0.067
Picture naming	Latency	*F*[1;38] = 0.379 *p* = 0.770	*F*[1;38] = 3.370 *p* = 0.074	*F*[1;38] = 1.610 *p* = 0.212
Picture naming	Duration	*F*[1;38] = 2.696 *p* = 0.109	*F*[1;38] = 667.326 *p* < 0.001	*F*[1;38] = 3.437 *p* = 0.072
Sentence production	Accuracy	*F*[1;38] = 0.284 *p* = 0.597	*F*[1;38] = 0.307 *p* = 0.583	*F*[1;38] = 10.591 *p* = 0.002
Sentence production	Latency	*F*[1;38] < 0.001 *p* = 0.984	*F*[1;38] = 3.370 *p* = 0.074	*F*[1;38] = 1.812 *p* = 0.186
Sentence production	Duration	*F*[1;38] = 9.779 *p* = 0.003	*F*[1;38] = 0.685 *p* = 0.413	*F*[1;38] = 0.829 *p* = 0.368
Discourse	TIU	*F*[1;38] < 0.001 *p* = 0.998	*F*[1;38] = 2.964 *p* = 0.093	*F*[1;38] = 16.589 *p* < 0.001
Discourse	LIU	*F*[1;38] = 3.247 *p* = 0.080	*F*[1;38] = 0.168 *p* = 0.668	*F*[1;38] = 2.184 *p* = 0.148

The set of 2 × 2 ANOVAs for **verbal fluency** revealed a main effects of *Condition* in the switch but not in the simple condition. Main effects of *Order* were not significant. The interaction term was significant for the Simple but not for the Switch condition. Fewer words were generated under the mimicked hallucinations than under noise.

The follow-up 2 × 2 × 2 ANOVA **combining the** two analyses for the **Simple and Switch variants of verbal fluency** into one large analysis yielded the following findings: In line with the differential pattern in the preceding analyses of the individual tasks, there was a significant interaction of *Task* and *Condition* (*F*[1;38] = 5.100, *p* = 0.030). The other effects were as follows. There was a main effect for *Task* (*F*[1;38] = 28.930, *p* < 0.001) but not for *Order* (*F*[1;38] = 0.325, *p* = 0.572) or *Condition* (*F*[1;38] = 2.177, *p* = 0.148). The interaction of *Order* with *Task* was significant (*F*[1;38] = 51.557, *p* < 0.001), but not the interaction of *Order* with *Condition* (*F*[1;38] = 0.061, *p* = 0.806). Finally, the 3-way interaction was significant (*F*[1;38] = 35.916, *p* < 0.001).

The set of 2 × 2 ANOVAs for **picture naming** revealed no significant main effects of *Condition* A main effects of *Order* was found for Accuracy and Duration, but not for Latency. Interaction terms were not significant. As accuracy did not differ between the two conditions, no additional qualitative analysis of the individual speech errors was performed.

The set of 2 × 2 ANOVAs for **sentence production** revealed a main effect of *Condition* for Duration but not for Accuracy or Latency. Main effects of *Order* were not significant. Interaction terms significant for Accuracy but not for Latency or Duration. Sentences under auditory-verbal hallucinations were spoken more quickly than in the noise condition. Again, as accuracy did not differ between the two conditions, no additional qualitative analysis of the individual speech errors was performed.

The set of 2 × 2 ANOVAs for **discourse** revealed no main effects of *Condition* or *Order*. The interaction term was significant for TIU but not for LIU.

**Figure 2:**
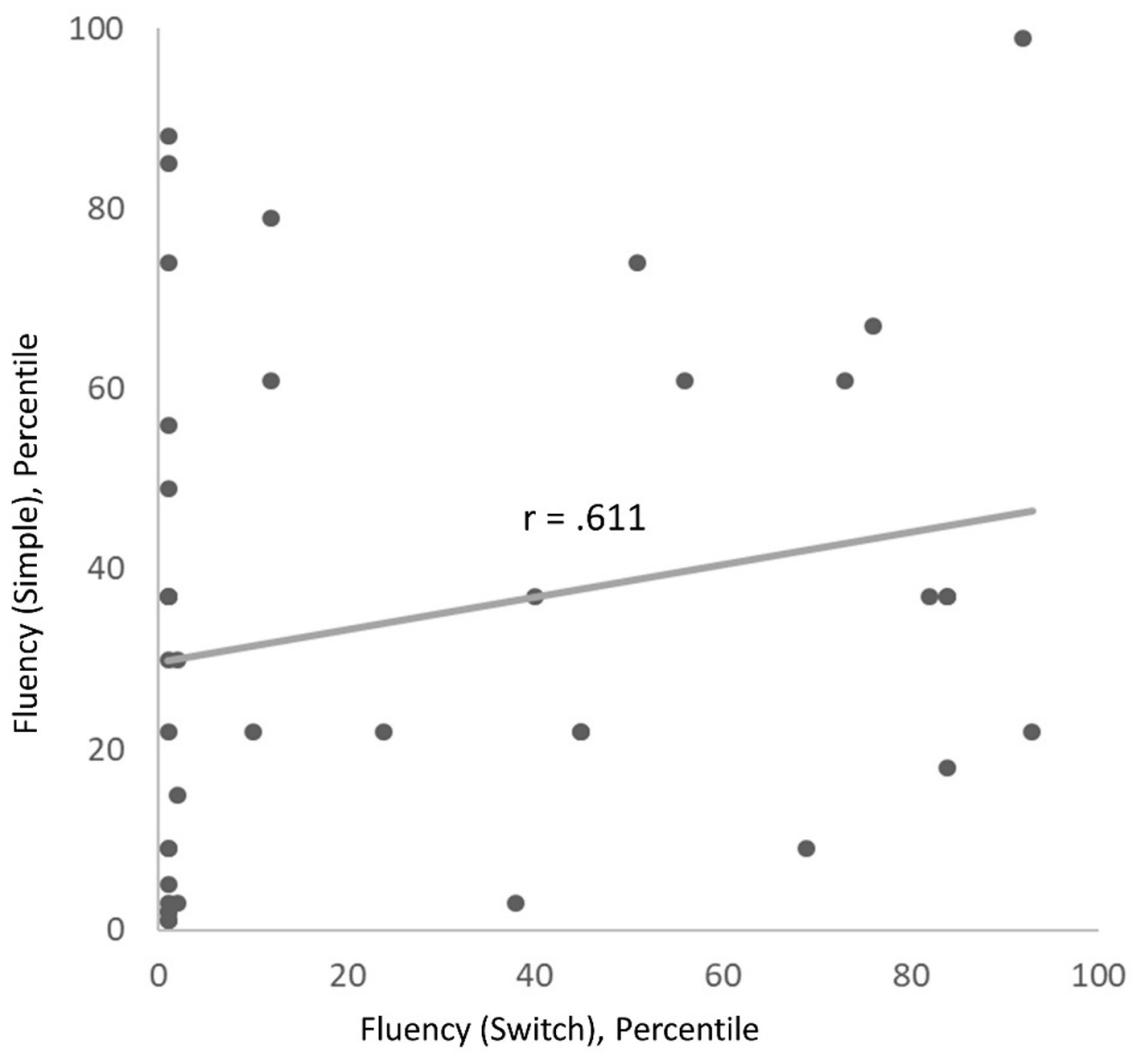
Scatter plot of the individual performance under mimicked auditory verbal hallucinations in the simple (y-axis) and the switch (x-axis) conditions of the verbal fluency task.

Finally, a Pearson correlation analysis looked into the potential relationship of the significant *Condition* effects for the verbal fluency *Switch* condition and the sentence production *Duration*. The difference effects for *Condition* (hallucination vs. noise) for verbal fluency *Switch* and sentence production *Duration* were not correlated (*r* = 0.189; *p* = 0.243). Looking at the individual correlation effects of each simulation variant (hallucination, noise) in each task (fluency Switch, fluency Simple), a significant correlation of the sentence duration under both conditions emerged (*r* = 0.611; *p* < 0.001; Figure 2). The other correlations were not significant (sentence_Duration_hallucination × fluency_Switch_hallucination: *r* = −0.206; *p* = 0.203; sentence_Duration_hallucination × fluency_Switch_noise: *r* = 0.001; *p* = 0.996; fluency_Switch_hallucination × fluency_Switch_noise: *r* = 0.240; *p* = 0.136). Please note that, even though no value of p correction for multiple comparisons was applied, the one significant effect would remain significant even after a strict Bonferroni correction.

## Discussion

The present study investigated the influence of mimicked auditory-verbal hallucinations (vs. noise) on different aspects of language production at the word, sentence, and text level. There was a significant influence of mimicked auditory-verbal hallucinations on single word production only in the most difficult task (fluency, switch condition). The distinction of the two fluency variants was confirmed by the significant interaction effect of *Task* and *Condition*. Moreover, sentences under mimicked hallucinations were spoken more rapidly than under noise. These effects of the simulation at word vs. sentence level seem to be of differential nature as there was no correlation whatsoever across linguistic levels.

These results indicate that the presence of mimicked auditory-verbal hallucinations, i.e., a linguistically meaningful source of distraction, can exert influence on the language production process over and above that of mere background noise, as in the Lombard effect which predominantly concerns the loudness and perhaps pitch of the speaker’s voice. Moreover, the overall pattern of results is largely in line with reports in the literature about the linguistic performance of real PwS with auditory-verbal hallucinations. For single word production, there is ample evidence for deficits in verbal fluency (e.g., Hintze et al. 2022; Wei et al., 2022; for a review *cf.* the meta-analysis by Doughty and Done, 2009). These verbal fluency deficits must be distinguished from a rather fair performance in simpler tasks of lexical retrieval such as picture naming (e.g., Al-Uzri et al., 2004; Tan and Rossell, 2017; but see Vogel et al., 2009). Thus, rather than assuming the locus of the effect (only) in lexical access *per se* (e.g., Heim, 2020), a link to other cognitive functions such as executive functions (e.g., working memory; processing speed) must be considered as potential causes for these specific deficits in schizophrenia (Ojeda et al., 2008; Berberian et al., 2016), as these functions are associated with verbal fluency and cognitive reserve in neurotypical persons and (at least in male) PwS (Marsh et al., 2019; Kubota et al., 2022). Also, the dissociation of verbal fluency deficits but no speech-onset latency effects in sentence production is in line with observations in real PwS vs. healthy controls (Creyaufmüller et al., 2020). Finally, the increases talking speed under mimicked hallucinations in the present study could be associated with reports of logorrhoea and concurrent hallucinations (Lee, 2004), a set of symptoms responding to the dosage of Lorazepam.

To conclude, the simulation paradigm employed here caused specific effects with the following properties: (1) They go beyond those of the presence of white noise; (2) They reflect findings in the literature about symptoms in real auditory-verbal hallucinations; (3) Over and above these reports in the literature, the effects observed here have a clear direction of causality: the auditory stimulation caused the speech effects and not vice versa (as was previously investigated in studies looking for the source of hallucinations in the impaired speech-output monitor).

The simulation applied here did not cause any further deficits in the tasks of connected speech (sentence production, discourse). This lack of effects was unexpected (*cf.* e.g., the systematic study by Vogel et al., 2009 that did report more errors in a sentence production task for PwS). Also, the differential pattern for deficits in the switch condition of the fluency task but not in the simple condition is not immediately reflecting the state-of-the-art: PwS tend to be affected already in the simpler version of the task. However, they are certainly affected in the version with category switches (Vogel et al., 2009; Creyaufmüller et al., 2020). For the sentence condition, one explanation could be that the type of sentences was too simple to be sensitive to the simulation, as it were only main clauses of the S-P-O type. Very little syntactic planning was necessary here – perhaps too little (Moro et al., 2015; see Creyaufmüller et al., 2020 for similiarly simple sentences with similar effects). Likewise, in the discourse task, the challenge may have been too easy since all pictures were presented for the full amount of time, and in the correct order: very much structure was provided so that the lexical and thematic processing (which was analysed here in terms of LIU and TIU) had enough context (see Kuperberg, 2010b) on the role and relevance of context for linguistic processing in schizophrenia.

This, however, is exactly the advantage of a simulation study, as outlined in the introduction: one can derive novel hypotheses which can be tested with healthy volunteers and, later, with people with schizophrenia. Two alternative hypotheses present themselves: (1) The mimicking method is not strong enough to produce meaningful effects beyond the single word level. – (2) Auditory-verbal hallucinations have a differential impact on language processing at the different linguistic levels: they interfere with lexical retrieval under cognitively demanding conditions but are less relevant for connected speech (leading to the next question which other pathological mechanisms, then, are interfering at the level of connected speech). Both alternatives can be pursued, (1) by using less structured materials and different methods of speech elicitation, and (2) by systematically juxtaposing the statistical effect of severity of auditory-verbal hallucinations in PwS with other carefully obtained and well-structured clinical scales and measures of cognitive and linguistic functioning. Such insights might help formulate more complex models of language processing that take into account processing levels beyond (psycho-)linguistics.

In any case, this study is among the first to suggest a potential causal link between auditory-verbal hallucinations and (a part of the) linguistic problems in language production in people with schizophrenia instead of the other way around. With the first preliminary knowledge obtained here, more far-reaching future studies, both behaviourally and in combination with neuroimaging, become feasible. Kuperberg (2010b, p: 600) asked, *“Is it possible to neuroanatomically dissociate language networks engaged in different types of psycholinguistic operations? How and at what stage do these networks interact? Are these neural mechanisms reciprocally linked such that an over-engagement of one network is necessarily accompanied by an under-engagement of another? The study of language processing in a widespread functional disorder such as schizophrenia may help us begin to address some of these questions.”*

In future studies, one aspect which might be considered is that of a potential habituation to the auditory stimulation during the tasks. We controlled for order effects by counter-balancing the occurrence of the mimicked hallucinations and that of white noise across participants. Statistically, the order effects were prominent in many cases (even though the actual experimental manipulation was strong enough to cause detectable effects). Also, subsequent studies might skip the picture naming task and perhaps start immediately with those tasks requiring longer and more complex connected speech, thus taking advantage of the simulation while habituation is still low. Finally, future studies might assess the degree of proneness to auditory hallucinations, as this may significantly modulate the amount to which the presence of hallucination-like stimuli actually affects behavior (*cf.* the recent study by Laloyaux et al., 2022).

### Open issues

In this study, the experimental condition contained mimicked auditory verbal hallucinations, i.e., verbal materials. These might *per se* have a higher salience than the non-verbal noise in the control condition (Rossell and Boundy, 2005; de Boer et al., 2019), so that distraction, i.e., empirically induced interference with cognitive control mechanisms such as attention, inhibition and also working memory, might have contributed to the differential effects in the verbal fluency tasks. We cannot rule out this possibility, which can be investigated more systematically in subsequent studies. But even if the experimental condition interacted with executive functions, this would be very much in line with the processes that potentially interfere with language processing in PwS. As outlined in the introduction, several studies have demonstrated the role of reduced executive functioning (cognitive control, attention) for the occurrence of real auditory-verbal hallucinations (e.g., Hugdahl, 2009; Hugdahl et al., 2013; Løberg et al., 2015; Waters et al., 2012). Also, the well-documented problems of PwS in verbal fluency tasks have been attributed to reduced executive functions, as discussed above. More generally, executive functions are essential also for language production aside from verbal fluency tasks (Sikora et al., 2016; San José et al., 2021; see Ye and Zhou, 2009, for a review), not only in PwS, but in every speaker. If now the presence of mimicked hallucinations had a distracting impact on the participants in the present study, thus affecting executive functions, this, in turn, might affect the controlled production of language (In fact, in the study by Kim and Wojnar (2019) that investigated the effects of mimicked auditory verbal hallucinations, the self-reports of their healthy volunteers suggest a significant amount of distraction, leading to problems with concentration and memory). A similar mechanisms might be assumed for the case of PwS: auditory-verbal hallucinations occur because of executive deficits, which also interfere with language production. In this explanation, the auditory-verbal hallucinations would thus not exert a direct influence on the language system, but would have an indirect effect *via* executive functions. Which model better accounts for the effects may be investigated in future studies.

### Limitations

The findings should be viewed in the light of the following potential limitations. First, as pointed out above, people may vary with respect to their proneness to auditory hallucinations, which is thus a potential source of noise in the present data. People who are potentially less prone to auditory hallucinations might also have a stronger sense of distance to them and consider them as mere auditory noise unrelated to themselves. Also, the effects might vary with the volume at which the auditory stimuli are presented. This, again, was only adjusted such that it was not unpleasant for the participants, but a systematic variation was not applied. Finally, it is possible that different contents (commenting vs. imperative, threatening vs. neutral) have different effects on language production. This question was not within the scope of the present study. Here, we wanted to test the influence of mimicked auditory-verbal hallucinations in a number of speaking tasks of different complexity – and of different duration. We kept the duration so short that all tasks fitted into one session. Moreover, the duration of the fluency tasks was dictated by the instructions of that tests. Otherwise we would not have been able to refer to the normative test values to keep the various variants parallel with respect to difficulty. Accordingly, we could not analyse the data with respect to the contents of the hallucinations.

## Conclusion

Based on earlier evidence for the usefulness of employing simulations to model language performance in patient samples, this study demonstrates that mimicking auditory-verbal hallucinations provokes language symptoms comparable to those reported in the literature. A refinement of the methods is suggested. In future, the simulation approach might help piloting studies with real patient samples, allowing more precise manipulations and thus increasing the impact of clinical studies with real PwS. In particular, the simulation may help formulating more detailed hypotheses about the (neuro-)cognitive foundations of the first-rank symptoms in schizophrenia and their interplay with the human cognitive apparatus.

For clinical purposes, the present findings might have implications for speech-language therapy and/or cognitive-behavioural therapy. For instance, they might inspire the development of meta-cognitive strategies of control over one’s own utterances (including semantic content and syntactic structure) in the presence of distracting auditory verbal hallucinations: these could be tested and evaluated in neurotypical participants hearing mimicked auditory-verbal hallucinations before application to PwS. Success in the management of one’s own voice and communication behaviour might be one key feature to interrupt the vicious circle in which PwS find themselves trapped (Heim, 2020): neurobiological alterations in the brain affect directly and indirectly (*via* auditory-verbal hallucinations) communication success, both in the personal life (reduced social participation) and in the psychotherapeutic setting (confuse or diffuse interaction with the therapist). Even if the neurobiological basis cannot be changed pharmaceutically, or if this change progresses slowly, improved control over one’s utterances despite hallucinations might sooner lead to more efficient communication, and hence improved participation and therapeutical exchange and, in consequence, quality of life (Joyal et al., 2016; Heim, 2020). “The field of SLT can contribute at characterizing speech and language impairments across life span of patients with schizophrenia in a detailed manner, as well as identifying and developing efficient approaches to treat speech and language impairments with evidence based data.” (Joyal et al., 2016, p. 93). In their review, Joyal et al. (2016) demonstrated the potential usefulness of various therapeutic approaches for improving language and communication skills, in particular pragmatic skills, in PwS, indicating that language abilities can really be modified by interventions. At the same time, the large heterogeneity of these approaches (operant conditioning; meta-comprehension/meta-learning; cognitive therapy; rehabilitative approaches), their settings (group or individual), and their parameters (duration of session: 15–90 min; total duration of intervention: 2 weeks up to 2 years; frequency of therapy 1x per week up to 2x per day) also illustrates that there is a large need for setting standards and for model-guided interventions. In a most recent RCT, Bambini et al. (2022) demonstrated that their novel approach PragmaCom, which included both production and comprehension elements, improved pragmatic communication skills of PwS after 12 weeks of training and also after a 3-months follow-up. As such RCTs can only be run after sufficient piloting, the paradigm used in the present study might prove useful for such piloting of intervention strategies in neurotypical persons before the most promising ones are actually applied to PwS, thus improving their chances to benefit from the trial they enrol in.

## Data availability statement

The raw data supporting the conclusions of this article will be made available by the authors, without undue reservation.

## Ethics statement

The studies involving human participants were reviewed and approved by Ethikkommission an der Medizinischen Fakultät der RWTH Aachen. The patients/participants provided their written informed consent to participate in this study.

## Author contributions

SH: study concept and design, supervision of the creation of materials/stimuli, data analysis and supervision of data analysis, discussion and manuscript. SP: creation of materials/stimuli, recruitment of participants, data acquisition, data analysis, and contribution to and revision of the manuscript. KH: study concept and design, supervision of the creation of materials/stimuli, supervision of the linguistic aspects of data analysis, discussion of statistical data analysis and results, and revisions of the manuscript. All authors contributed to the article and approved the submitted version.

## Funding

This work is funded by the Deutsche Forschungsgemeinschaft (DFG, German Research Foundation) – 491111487.

## Conflict of interest

The authors declare that the research was conducted in the absence of any commercial or financial relationships that could be construed as a potential conflict of interest.

## Publisher’s note

All claims expressed in this article are solely those of the authors and do not necessarily represent those of their affiliated organizations, or those of the publisher, the editors and the reviewers. Any product that may be evaluated in this article, or claim that may be made by its manufacturer, is not guaranteed or endorsed by the publisher.

## Footnotes

^1^ http://www.dlexdb.de/

^2^ https://www.ibm.com
