# Supplementary material for: Mimicking effects of auditory verbal hallucinations on language production at the level of words, sentences and stories
Source: Front Psychol. 2022 Nov 16;13:1017865. doi: 10.3389/fpsyg.2022.1017865 (PMC9709433; doi:10.3389/fpsyg.2022.1017865)
Supplement: Supplementary file 1 [file Table_2.pdf]

Table 2: Verbatim transcripts (German original and English translation via Google Translate: https://translate.google.com) of the three types of audio-verbal hallucinations, used as stimuli in the present study. Please enter the transcript into automated translation algorithms like Google Translate in order to obtain drafts for use in other languages after perhaps some additional revisions.

Type of voice	*Person*	
Commenting	P1: Heute ist so ein schöner, sonniger Tag.<br>Alle sind draußen und genießen das Wetter.<br>Und du, sitzt allein in deiner Wohnung fest.<br>Liegst auf dem Sofa, guckst Fernsehen?!<br>Da läuft immer dasselbe.<br>Immer um die gleiche Uhrzeit.<br>Hier wurde auch schon länger nicht mehr aufgeräumt.<br>Auf dem Boden liegen Klamotten.<br>Und in der Küche - ist alles voller Geschirr. Dreckiges Geschirr.<br>Hm, wie lange es da wohl schon liegt?<br>Und Staub wischen könnte man hier auch mal.<br>Die Staubschicht ist kaum zu übersehen.<br>Hier kann man keinen Besuch empfangen.<br>Ich würde mich hier nicht wohlfühlen.<br>Hm, aber das Sofa ist auch schön gemütlich.<br>Das Geschirr kann man auch morgen abwaschen.<br>Es kommt heute eh keiner mehr.	P1: Today is such a beautiful, sunny day.<br>Everyone is outside enjoying the weather.<br>And you, stuck in your apartment alone.<br>Lying on the sofa, watching TV?!<br>It's always the same thing.<br>Always at the same time.<br>It hasn't been cleaned up here for a long time.<br>There are clothes on the floor.<br>And in the kitchen - everything is full of dishes. Dirty dishes.<br>Hm, how long has it been there?<br>And you could wipe the dust here too.<br>The layer of dust is hard to miss.<br>Visitors are not allowed here.<br>I wouldn't feel comfortable here.<br>Hm, but the sofa is also nice and cozy.<br>You can wash the dishes tomorrow too.<br>Nobody's coming today anyway.
Dialogic	P2: Als ob es morgen sauberer aussehen wird? So sieht es hier doch immer aus.<br>P1: Das stimmt nicht! Wenn er aufräumt, ist es hier doch ordentlich.<br>P2: Wann passiert das schon? Er ist ein faules Stück.<br>P1: Manchmal kann man sich ja wohl ausruhen. Dann ist man ja nicht direkt faul.<br>P2: Manchmal? Der ruht sich nur aus. Wie lange hat er heute wieder geschlafen?<br>P1: Er wird nun mal schnell müde.<br>P2: Wovon denn? Wenn man nichts tut, kann man auch nicht müde werden.	P2: As if it will look cleaner tomorrow? That's how it always looks here.<br>P1: That's not true! If he cleans up, it's tidy here.<br>P2: When will that happen? He's a lazy piece.<br>P1: Sometimes you can rest well. Then you're not exactly lazy.<br>P2: Sometimes? He's just resting. How long did he sleep again today?<br>P1: He gets tired quickly.<br>P2: Of what? If you don't do anything, you can't get tired.<br>P1: We also distract quite a bit at night.

	P1: Wir lenken ja nachts auch ganz schön ab.<br>P2: Nein, du lenkst ab. Du erzählst ja auch nur Blödsinn. Wenn er mal auf mich hören würde, wäre es hier schon längst sauber.<br>P1: Hör nicht auf ihn, er regt sich bloß darüber auf, dass du mich lieber magst.<br>P2: Wenn du weiter auf diese Quasselstrippe hörst, wirst du aus der Wohnung fliegen. Das ist ein riesen Messi-Haufen.<br>P1: Es reicht ja auch, wenn man die Wohnung morgen aufräumt. Das läuft nicht weg. Die Sendung ist doch gerade so spannend.	P2: No, you distract. You're just talking nonsense. If he would listen to me, it would have been clean here long ago.<br>P1: Don't listen to him, he's just upset that you like me better.<br>P2: If you keep listening to this chatterbox, you'll be kicked out of the apartment. That's a huge Messi bunch.<br>P1: It's enough if you tidy up the apartment tomorrow. This doesn't go away. The show is so exciting right now.
Imperative	P2: War ja klar, dass du mal wieder nichts auf die Reihe bekommst.<br>Immer liegst du nur faul auf dem Sofa rum.<br>Nicht ein Mal duschen schaffst du dich.<br>Spürst du diesen Gestank? Das bist du!<br>Ja das kommt davon, wenn man sich 7 Tage am Stück nicht wäscht.<br>Nichts schaffst du, nicht einmal das.<br>Denkst du irgendjemand will mit einem Stinktier befreundet sein?<br>Geschweige denn so jemanden lieben?<br>Niemand! Niemand wird dich lieben.<br>Deshalb haben sich alle von dir abgewendet.<br>Du bist ekelhaft.<br>Du bist nicht liebenswert.<br>Und weißt du was? Das ist alles deine eigene Schuld<br>Wieso lässt du sich so gehen?<br>Wann hast du das letzte Mal in den Spiegel geschaut?<br>Obwohl - tu es besser nicht, sonst erschreckst du dich vor dir selbst.<br>Mir wird ja schon übel, wenn ich dich anschaue.<br>Das will keiner sehen … sowas.<br>Du solltest dich schämen.<br>Niemals würde ich so auf die Straße gehen.<br>Nicht mit so einem Aussehen.	P2: It was clear that you wouldn't get anything done again.<br>You always just lie around lazily on the sofa.<br>You can't even take a shower.<br>Do you feel that stench? You are that!<br>Yes, that's what happens when you don't wash for 7 days straight.<br>You can't do anything, not even that.<br>Do you think anyone would want to be friends with a skunk?<br>Let alone love someone like that?<br>No one! nobody will love you<br>That's why everyone turned their backs on you.<br>You are disgusting.<br>You are unlovable.<br>And you know what? This is all your own fault<br>Why are you letting yourself go like this?<br>When was the last time you looked in the mirror?<br>Although - better not do it, otherwise you will scare yourself.<br>I get sick just looking at you.<br>Nobody wants to see that... something like that.<br>You should be ashamed of yourself.<br>I would never walk the streets like that.<br>Not with that look.

Commenting	P2: Irgendwie ist es etwas frisch geworden.<br>Vielleicht sollte man das Fenster schließen.<br>Hier wurde ja auch schon lang genug gelüftet.<br>Huch, die Gardine hat sich im Fenster verfangen.<br>Hoffentlich ist sie nicht gerissen.<br>So ganz passt die Farbe der Vorhänge aber nicht zu den Möbeln.<br>Braune Vorhänge und eine graue Couch?<br>Naja, nächstes Mal sollte man sich vielleicht beraten lassen.<br>Oh ja, und die Fenster hätten es auch mal nötig geputzt zu werden.<br>Hm, wurden sie eigentlich jemals geputzt?<br>Vielleicht von dem Vormieter.<br>Na immerhin.<br>Mensch, heute ist aber ganz schön viel los auf den Straßen.<br>So viele Autos, aber auch ganz viele Fahrradfahrer.<br>Von denen gibt es auch immer mehr.<br>Fahrrad fahren ist ja auch gut für die Figur.<br>Bei dem Wetter macht das ja auch Spaß.<br>Aber im Winter wäre das zu kalt und zu nass.<br>Das kann ja auch schnell gefährlich werden.	P2: Somehow it got a little fresh.<br>Maybe you should close the window.<br>It's been aired out here long enough.<br>Oops, the curtain got caught in the window.<br>I hope she isn't torn.<br>The color of the curtains doesn't quite match the furniture.<br>Brown curtains and a gray couch?<br>Well, next time you might want to seek advice.<br>Oh yes, and the windows needed cleaning too.<br>Hm, were they actually ever cleaned?<br>Maybe from the previous tenant.<br>Well at least.<br>Man, there's a lot going on on the streets today.<br>So many cars, but also a lot of cyclists.<br>There are more and more of them.<br>Riding a bike is also good for your figure.<br>It's fun when the weather is good.<br>But in winter that would be too cold and too wet.<br>That can quickly become dangerous.
Dialogic	P1: Dir würde es auch nicht schaden, etwas mehr Sport zu treiben. Sonst wird das nichts mit der Sommerfigur.<br>P2: Der Sommer ist doch schon da. Dafür ist es doch eh zu spät. Außerdem geht es sowieso nicht in den Strandurlaub.<br>P1: Mal wieder. Wann hast du das letzte Mal einen Strand gesehen?<br>P2: Manche fahren jedes Jahr in den Urlaub und er war schon mehrere Jahre nicht weg.<br>P1: Ein Urlaub kostet ja auch viel Geld.<br>P2: Ja, wenn man ein fauler Nichtsnutz ist, dann kann man ja auch kein Geld verdienen.<br>P1: Ich würde so gerne Mal auf die Malediven fliegen.	P1: It wouldn't hurt you to do a little more sport either. Otherwise it won't work with the summer figure.<br>P2: Summer is already here. It's too late for that anyway. Besides, it's not going on a beach vacation anyway.<br>P1: Again. When was the last time you saw a beach?<br>P2: Some go on vacation every year and he hasn't been away for several years.<br>P1: A vacation also costs a lot of money.<br>P2: Yes, if you're a lazy good-for-nothing then you can't make any money either.<br>P1: I would love to fly to the Maldives.<br>P2: Maldives? You only fly there for the honeymoon.

	P2: Malediven? Da fliegt man doch nur über die Flitterwochen hin.<br>P1: Dann passiert das ja nie. Wann hattest du das letzte Mal eine Freundin?<br>P2: Man muss ja auch rausgehen, um jemanden kennenzulernen.<br>P1: Heutzutage geht das alles über Dating-Apps.<br>P2: Muss man da nicht auch Fotos von sich rausstellen?<br>P1: Ja, aber die guten Fotos sind schon alle alt. Es müssen Neue her.<br>P2: Momentan sollten lieber keine Fotos von dir geschossen werden.	P1: Then that never happens. When was the last time you had a girlfriend?<br>P2: You have to go out to get to know someone.<br>P1: It's all about dating apps these days.<br>P2: Don't you have to put out photos of yourself as well?<br>P1: Yes, but the good photos are all old. New ones are needed.<br>P2: It would be better not to take any photos of you at the moment.
Imperative	P1: Wer würde denn schon Fotos von dir machen wollen?<br>Siehst du denn nicht, dass alle sich von dir abgewendet haben!<br>Du bist ein langweiliges, nichtsnutziges Häufchen Elend.<br>Derjenige, der nichts auf die Reihe bekommt.<br>Schule abgebrochen, Ausbildung abgebrochen.<br>Alles abgebrochen – nichts kannst du Mal durchziehen.<br>Und weißt du wieso? Weil du einfach zu dumm dafür bist.<br>Du bist einfach zu dumm, um dir die ganzen Lerninhalte zu merken.<br>Guck mal, alle kriegen es hin – nur du nicht.<br>In deinem Alter haben alle schon was erreicht.<br>Ein abgeschlossenes Studium, Haus und Kind.<br>Und du? Hockst hier allein in deiner verdreckten Bude rum.<br>Isst den ganzen Tag nur Müll und wirst immer fetter.<br>Aus dir wird nie was werden.<br>Deshalb hassen dich deine Eltern auch.<br>Sie haben einen guten Sohn erzogen und was ist aus dir geworden?<br>Du enttäuscht bloß alle. Du bist die reinste Enttäuschung.<br>Deine Familie wäre glücklich, wenn es dich nicht gäbe.<br>Endlich wäre das Sorgenkind weg.<br>Alle Probleme wären weg.<br>Nicht nur ihre – sondern deine Probleme – sie wären auch weg.	P1: Who would want to take pictures of you?<br>Can't you see that everyone has turned their backs on you!<br>You're a boring, useless heap of misery.<br>The one who can't get anything done.<br>Dropped out of school, dropped out of college.<br>Everything canceled - you can't go through with anything.<br>And do you know why? Because you're just too stupid for that.<br>You're just too stupid to remember all the learning content.<br>Look, everyone can do it - just not you.<br>At your age, everyone has already achieved something.<br>A college degree, home and kids.<br>And you? Sitting here alone in your filthy place.<br>Eats junk all day and keeps getting fatter.<br>You will never become anything.<br>That's why your parents hate you too.<br>They raised a good son and what became of you?<br>You just disappoint everyone.<br>You are a total disappointment.<br>Your family would be happy if it weren't for you.<br>Finally the problem child would be gone.<br>All problems would be gone.<br>Not only theirs - but your problems - they would be gone too.

Commenting	P2: Du gehst in Richtung Badezimmer.<br>Du schaust dich im Spiegel an.<br>Erstmal rasierst du dich, dann putzt du dir die Zähne.<br>Das sieht doch schon viel besser aus.<br>Eine schöne Dusche nehmen, die Haare waschen, ein paar frische Klamotten anziehen und schon bist du fast wie neu.<br>Nun machst du dich auf den Weg zum Friseur.<br>Hoffentlich nimmt er dich auch ohne Termin dran.<br>Am besten noch ein Foto von der Wunschfrisur raussuchen.<br>Zuerst aber nicht vergessen die Tür abzuschließen.<br>Handy, Portmonee und Schlüssel sind dabei.<br>Für eine Busfahrkarte hast du genug Geld eingepackt.<br>Die Fahrt wird jetzt 20 Minuten dauern.<br>Der Bus ist aber ziemlich voll.<br>Du musst erstmal stehen, weil der Bus so überfüllt ist.<br>Hinten schreit ein Baby, das ist ganz schön nervig.<br>Seine Mutter ist aber nur mit ihrem Handy beschäftigt.<br>Was für eine Rabenmutter.<br>Du magst Busfahrten nicht, immer dieser unangenehme Körperkontakt mit Fremden.<br>Hoffentlich steigen gleich schon welche aus.<br>Dann kannst du dich hinsetzten.<br>Wann hört das Baby endlich auf zu schreien?<br>Dich macht die ganze Situation hier nervös.<br>Wieso bist du nicht auf der Couch geblieben?	P2: You walk towards the bathroom.<br>You look at yourself in the mirror.<br>First you shave, then you brush your teeth.<br>That looks a lot better.<br>Take a nice shower, wash your hair, put on some clean clothes and you're almost brand new.<br>Now you make your way to the hairdresser.<br>Hopefully he'll take you without an appointment.<br>It is best to find a photo of the desired hairstyle.<br>But first, don't forget to lock the door.<br>Mobile phone, wallet and keys are included.<br>You packed enough money for a bus ticket.<br>The journey will now take 20 minutes.<br>But the bus is quite full.<br>You have to stand first because the bus is so crowded.<br>A baby is screaming in the back, it's really annoying.<br>But his mother is only busy with her mobile phone.<br>What a raven mother.<br>You don't like bus rides, always that awkward physical contact with strangers.<br>Hopefully some will get off soon.<br>Then you can sit down.<br>When will the baby finally stop crying?<br>The whole situation here makes you nervous.<br>Why didn't you stay on the couch?
Dialogic	P1: Hast du diese Frau im Bus nicht eben auf der Straße gesehen?<br>P2: Sie war auch auf der Haltestelle.<br>P1: Ich glaube sie verfolgt ihn.<br>P2: Ja, sie guckt die ganze Zeit zu ihm rüber.<br>P1: Sie wendet immer den Blick ab, wenn sie sieht, dass er zurückguckt.<br>P2: Ich glaube sie beobachtet ihn. Was kann sie nur wollen?	P1: Didn't you just see that woman on the bus on the street?<br>P2: She was also at the station.<br>P1: I think she's following him.<br>P2: Yes, she looks over at him all the time.<br>P1: She always looks away when she sees him looking back.<br>P2: I think she's watching him. What can she possibly want?<br>P1: Maybe she wants to steal his money.

	P1: Vielleicht will sie sein Geld stehlen.<br>P2: Oder vielleicht sein Handy? Sie hat eben genau geschaut, wo er es reingelegt hat.<br>P1: Am besten du setzt dich an das andere Ende des Busses.<br>P2: Und gut auf das Handy aufpassen!<br>P1: Das Handy ist doch sowieso uralt, das will doch keiner haben.<br>P2: Das weißt sie ja nicht. Sie ist bestimmt eine Diebin. Sie will es stehlen und dann weiterverkaufen.<br>P1: Schau mal hin, sie hat sich auch umgesetzt. Sie verfolgt ihn wirklich.<br>P2: Also, wenn sie gleich auch noch an der gleichen Haltestelle aussteigt, hat sie es garantiert auf ihn abgesehen.<br>P1: Ich glaube der Friseurbesuch ist keine gute Idee. Zumindest heute nicht.<br>P2: Ja, am besten schnell aussteigen und nach Hause gehen.<br>P1: Hoffentlich läuft sie ihm nicht hinterher.	P2: Or maybe his cell phone? She just checked where he put it.<br>P1: It's best if you sit at the other end of the bus.<br>P2: And watch out for your cell phone!<br>P1: The mobile phone is very old anyway, nobody wants it.<br>P2: You don't know that. She must be a thief. She wants to steal it and then resell it.<br>P1: Take a look, she has also implemented it. She's really following him.<br>P2: Well, if she gets off at the same stop, she's definitely after him.<br>P1: I don't think going to the hairdresser's is a good idea. At least not today.<br>P2: Yes, it's best to get out quickly and go home.<br>P1: I hope she doesn't run after him.
Imperative	P2: Die Frau hat es auf dich abgesehen.<br>Renn so schnell wie es geht weg. Sie ist böse.<br>Sie will dir was Böses antun.<br>Sie will dich beklauen.<br>Nachdem sie all‘ dien Geld und all‘ deine Wertsachen gestohlen hat, wird sie versuchen dich umzubringen.<br>Du musst dich schützen. Du musst dein Schloss auswechseln.<br>Und du brauchst etwas, um dich zu wehren.<br>Du musst eine Waffe besorgen oder ein Messer.<br>Nachdem sie dich umgebracht hat, wird sie deine Familie ausfindig machen.<br>Und deine Eltern umbringen.<br>Ihr seid alle in Gefahr!<br>Aber es gibt eine Lösung.<br>Es gibt eine Möglichkeit sich zu retten.<br>Du musst ihr zuvorkommen.<br>Du musst schneller sein als sie.	P2: The woman is after you.<br>Run away as fast as you can.<br>she is evil<br>She wants to do something bad to you.<br>She wants to steal from you.<br>After stealing all your money and valuables, she will try to kill you.<br>You have to protect yourself.<br>You need to change your lock.<br>And you need something to fight back.<br>You need to get a gun or a knife.<br>After she kills you, she will track down your family.<br>And kill your parents.<br>You are all in danger!<br>But there is a solution.<br>There is a way to save yourself.<br>You have to forestall her.<br>You have to be faster than them.<br>You have to warn everyone and then you have to attack them.<br>Buy that one gun or get a knife.

	Du musst alle warnen und dann musst du sie angreifen.<br>Kauf die eine Waffe oder hol dir ein Messer.<br>Und dann suchst du sie und bringst sie um, bevor sie es tut.<br>Wenn du es nicht macht, wenn du dich nicht traust, dann wird sie es tun.<br>Sie wird dir alles nehmen, was du hast.<br>Dein Geld, dein Handy, deine Familie, dein Leben.<br>Du musst ihr zuvorkommen!<br>Tu es! Tu es selbst! Sei schneller als sie!<br>Bevor es zu spät ist!	And then you find her and kill her before she does.<br>If you don't do it, if you don't dare, then she will.<br>She will take everything you have from you.<br>Your money, your phone, your family, your life.<br>You must forestall her!<br>Do it! Do it yourself! Be faster than them!<br>Before it is too late!
Commenting	P1: Du rennst, du rennst so schnell du kannst.<br>Als ob du, um dein Leben rennen würdest.<br>Der Schweiß fließt dir den Nacken runter.<br>Du rennst sogar über die rote Ampel.<br>Gut, dass gerade keine Autos kommen.<br>Die Menschen drehen sich nach dir um, aber du bleibst nicht stehen.<br>Völlig außer Atem bist du angekommen.<br>Du hast dich kein einziges Mal umgedreht.<br>Schnell schließt du die Haustür auf und stürmst in deine Wohnung.<br>Drei Mal den Schlüssel rumdrehen und schon ist die Tür verschlossen.<br>Niemand kann reinkommen.<br>Du bist nun in Sicherheit. Schweißgebadet, aber in Sicherheit.<br>So schnell wirst du keinen Bus mehr nehmen.<br>Du kontrollierst dein Handy und dein Portmonee.<br>Alles ist an seinem Platz. Du atmest erleichtert durch.<br>Nun räumst du dir einen Weg frei, um bis ins Wohnzimmer zu gelangen.<br>Dabei musst du über sämtliche Gegenstände drüber stolpern.<br>Deine Schuhe lässt du mitten im Weg stehen.<br>Und auch die Jacke landet auf dem Boden.<br>Neben den anderen Kleidungsstücken, die seit	P1: You run, you run as fast as you can.<br>As if you were running for your life.<br>The sweat runs down your neck.<br>You even run through the red light.<br>It's a good thing there aren't any cars coming.<br>People turn to you, but you don't stop.<br>You arrived completely out of breath.<br>You didn't turn around once.<br>You quickly unlock the front door and storm into your apartment.<br>Turn the key three times and the door is locked.<br>Nobody can come in.<br>You are safe now. Drenched in sweat but safe.<br>You won't be taking a bus anytime soon.<br>You control your cell phone and your wallet.<br>Everything is in its place. You breathe a sigh of relief.<br>Now you clear a way to get to the living room.<br>You have to stumble over all the objects.<br>You leave your shoes in the middle of the path.<br>And the jacket also lands on the floor.<br>Along with the other clothes that haven't been put away in weeks.

	Wochen nicht weggeräumt wurden.	
Dialogic	P2: Und wieder zurück in der dreckigen Bude, auf der Couch, so wie jeden Tag.<br>P1: Man sieht ja, was passiert, wenn man rausgeht. Da lauern nur Gefahren und Menschen, die einem was Böses wollen. Die Frau ist nicht die Einzige. Es gibt jede Menge böser Menschen.<br>P2: Wenigstens hat er ein bisschen das gute Wetter genießen können.<br>P1: Das nennst du genießen? Er wurde verfolgt und fast bestohlen. Hier ist er wenigstens in Sicherheit.<br>P2: Hier kann er den ganzen Tag faulenzen und weiterhin verblöden.<br>P1: Nein, schau mal, er wird gleich was kochen, statt auf der Couch zu liegen.<br>P2: Was wird es denn heute wieder Gesundes geben? Pizza, Nudeln oder eine Chipstüte?<br>P1: Eigentlich müsste er heute einkaufen gehen. Der Kühlschrank ist ziemlich leer.<br>P2: An seiner Stelle würde ich die Wohnung heute nicht mehr verlassen.<br>P1: Ja stimmt, die Frau aus dem Bus könnte vor der Tür auf ihn warten. Das ist zu gefährlich.<br>P2: Dann vielleicht etwas bestellen?<br>P1: Und wenn sie das Gespräch mithört?<br>P2: Dann wird sie wissen, dass er die Tür öffnen muss, wenn die Bestellung kommt.<br>P1: Nein, das ist zu gefährlich. Die Chips müssten für heute reichen.<br>P2: Ja, er kann ja auch morgen wieder einkaufen gehen.	P2: And back in the dirty shack, on the couch, like every day.<br>P1: You can see what happens when you go outside. Only dangers and people who want something bad are lurking there. The woman is not the only one. There are lots of bad people.<br>P2: At least he was able to enjoy the good weather a bit.<br>P1: You call that enjoying? He was followed and almost robbed. At least he's safe here.<br>P2: Here he can laze around all day and keep going stupid.<br>P1: No, look, he's about to cook something instead of lying on the couch.<br>P2: What will be healthy again today? Pizza, pasta or a bag of chips?<br>P1: Actually, he should go shopping today. The fridge is pretty much empty.<br>P2: If I were him, I wouldn't leave the apartment today.<br>P1: That's right, the woman from the bus could be waiting for him in front of the door. This is too dangerous.<br>P2: Then maybe order something?<br>P1: And if she overhears the conversation?<br>P2: Then she will know that he has to open the door when the order comes.<br>P1: No, that's too dangerous. The chips should be enough for today.<br>P2: Yes, he can go shopping again tomorrow.
Imperative	P1: Und mal wieder isst du nur ungesundes Fast Food.<br>Und wirst dicker und dicker.<br>Wie war das mit den guten Vorsätzen für dieses Jahr?<br>Sport und gesunde Ernährung?<br>Das war wir immer leeres Gerede.<br>Du schaffst eh nie etwas, was du dir vornimmst.	P1: And once again you only eat unhealthy fast food.<br>And get fatter and fatter.<br>How about the good intentions for this year?<br>Sport and healthy eating?<br>That was always idle talk.<br>You never achieve anything you set your mind to.<br>You just have no stamina, no willpower.

	Du hast einfach kein Durchhaltevermögen, keine Willenskraft.<br>Du wirst niemals abnehmen.<br>Du wirst immer das Dickerchen bleiben, dass in der Schule gemobbt wurde.<br>All‘ diese Beschimpfungen immer und das zurecht!<br>Du hast dich nie gewehrt. Du hast dich immer fertigmachen lassen.<br>Du bist einfach schwach, ein waschechtes Opfer.<br>Wieso tust du dir das alles eigentlich alles an?<br>Dies Leben? Dieses erbärmliche Leben?<br>Wieso stehst du jeden Morgen wieder auf?<br>Wofür kämpfst du?<br>Es lohnt sich sowieso nicht!<br>Du kannst dich nicht ändern, du wirst immer so bleiben, wie du bist.<br>So schwach und zu faul.<br>Für diese Schwäche musst du bezahlen.<br>Du musst dich bestrafen.<br>Dafür, dass du wieder gegen deine Vorsätze verstoßen hast.	You will never lose weight.<br>You'll always be the fat kid who was bullied at school.<br>All these insults always and rightly so!<br>You never fought back. You always let yourself be beaten up.<br>You're just weak, a real victim.<br>Why are you doing all of this to yourself?<br>This life? This pathetic life?<br>Why do you get up every morning?<br>what are you fighting for<br>It's not worth it anyway!<br>You can't change, you will always stay the way you are.<br>So weak and too lazy.<br>You have to pay for this weakness.<br>You have to punish yourself.<br>For breaking your resolutions again.
Commenting	P2: Und wieder liegst du die ganze Nacht wach.<br>Du kannst nicht schlafen.<br>Der Fernseher läuft immer noch.<br>Nachts laufen wirklich seltsame Sendungen.<br>Eigentlich wäre es besser ins Bett zu gehen.<br>Das Bett ist gemütlicher und auch bequemer.<br>Aber du hast keine Lust aufzustehen.<br>Dann musst du dir die Zähne putzen und dir einen Schlafanzug anziehen.<br>Das ist anstrengend.<br>Du schläfst doch meistens auf der Couch ein.<br>Daher kommen die Rückenschmerzen.<br>Du hast ein bisschen Hunger.<br>Vielleicht isst du einen Mitternachtssnack.<br>Kontrollier noch mal, ob die Tür abgeschlossen ist.<br>Jetzt bist du ja sowieso schon Mal aufgestanden.<br>Alles abgeschlossen.<br>Der Mond ist so hell. Er scheint in das Zimmer rein.	P2: And again you lie awake all night.<br>You can not sleep.<br>The TV is still on.<br>Really strange shows are on at night.<br>Actually, it would be better to go to bed.<br>The bed is cozier and also more comfortable.<br>But you don't feel like getting up.<br>Then you have to brush your teeth and put on your pajamas.<br>That is exhausting.<br>You usually fall asleep on the couch.<br>Hence the back pain.<br>You're a little hungry.<br>Maybe you eat a midnight snack.<br>Double check that the door is locked.<br>You're already up anyway.<br>All completed.<br>The moon is so bright. It shines into the room.<br>It's probably full moon. That's why you can't sleep.<br>It's three o'clock in the morning.

	Wahrscheinlich ist Vollmond. Deshalb kannst du nicht schlafen.<br>Es ist drei Uhr nachts.<br>Dir ist langweilig. Aber schlafen kannst du auch nicht.<br>Was läuft denn auf den anderen Kanälen?	You're bored. But you can't sleep either.<br>What's on the other channels?
Dialogic	P1: Neuer Tag, neues Glück. Auf dem Plan steht einkaufen zu gehen.<br>P2: Ja, es wäre Zeit für ein Frühstück…um 15 Uhr.<br>P1: Der Tag fängt natürlich später an, wenn man die ganze Nacht nicht schlafen konnte.<br>P2: Nicht, dass die Tage bei ihm jemals früher anfangen würden…<br>P1: Das liegt alles an dem Vollmond. Er war so hell, dass man nicht schlafen konnte.<br>P2: Na hoffentlich räumt er heute endlich mal die Wohnung auf.<br>P1: Erst einmal muss was gegessen werden.<br>P2: Jaja, Hauptsache der Fernseher ist schon an. Das kann ja nur schieflaufen.<br>P1: Der Fernseher kann doch im Hintergrund laufen. Das stört doch keinen.<br>P2: Guck mal, der ganze Einkaufswagen ist wieder voller Fast Food. Kauf doch bisschen Obst und Gemüse.<br>P1: Obst und Gemüse schmeckt aber nicht. Man muss essen, was man gerne mag.<br>P2: Der isst doch seit Jahren nur diesen Unsinn. Deshalb ist er ständig krank. Keine Abwehrkräfte.<br>P1: Ach was, in Pizza sind auch Vitamine drin. Guck mal, da sind Tomaten und Salat drauf.<br>P2: Wow! Und wie viele Fette sind da drin?<br>P1: Dein Gerede bringt nur schlechte Laune. Es ist großartig, dass er aufgestanden und in den Supermarkt gegangen ist.	P1: New day, new luck. The plan is to go shopping.<br>P2: Yes, it would be time for breakfast…at 3pm.<br>P1: Of course, the day starts later if you haven't been able to sleep all night.<br>P2: Not that his days ever start earlier...<br>P1: It's all because of the full moon. It was so bright you couldn't sleep.<br>P2: Well, I hope he finally tidies up the apartment today.<br>P1: First something has to be eaten.<br>P2: Yes, the main thing is that the television is already on. That can only go wrong.<br>P1: The television can run in the background. That doesn't bother anyone.<br>P2: Look, the whole shopping cart is full of fast food again. Buy some fruit and veg.<br>P1: But fruit and vegetables don't taste good. You have to eat what you like.<br>P2: He's only been eating this nonsense for years. That's why he's always sick. No defenses.<br>P1: Oh, there are vitamins in pizza too. Look, there are tomatoes and lettuce on it.<br>P2: wow! And how many fats are in there?<br>P1: Your talk only puts you in a bad mood. It's great that he got up and went to the supermarket.
Imperative	P2: Na, hörst du mich wieder?<br>Ich bin wieder da.<br>Nein, ich bin nicht weg.<br>Oder hast du dich etwas gefreut?	P2: Well, can you hear me again?<br>I'm back.<br>No, I'm not gone.<br>Or were you happy?<br>I will never disappear

	Ich werde niemals verschwinden.<br>Und du kannst nichts dagegen tun!<br>Wehe, du erzählst jemanden, dass du Stimmen hörst!<br>Obwohl - erzähl es!<br>Erzähl doch allen, dass du Stimmen hörst!<br>Dann sperren sie dich ein. Da, wo du auch hingehörst!<br>Natürlich bist du krank, was denn sonst?<br>Jaja, sie erzählen dir, dass du gesund werden kannst.<br>Dass du dich ändern kann!<br>Aber sie kennen dich nicht!<br>Sie kennen dich nicht so, wie ich dich kenne!<br>Du bist einfach zu blöd dafür!<br>Weißt du noch vor ein paar Tagen?<br>Als du Zigaretten aus dem Automaten kaufen wolltest?<br>Nicht einmal das hast du geschafft!<br>Dafür warst du selbst zu blöd!<br>Und jetzt glaubst du, dass du gesund werden kannst.<br>Dass du mich loswerden kannst!<br>Ha, dass ich nicht lache!<br>Du wirst mich niemals loswerden!<br>Ich werde erst verschwinden, wenn du niemals wieder aufwachst.	And you can't do anything about it!<br>Don't tell someone you hear voices!<br>Although - tell me!<br>Tell everyone you hear voices!<br>Then they lock you up. Where you belong too!<br>Of course you're sick, what else?<br>Yeah, they tell you that you can get well.<br>That you can change!<br>But they don't know you!<br>They don't know you like I know you!<br>You're just too stupid for that!<br>Do you remember a few days ago?<br>When you wanted to buy cigarettes from the machine?<br>You didn't even make it!<br>You were too stupid for that yourself!<br>And now you think you can get well.<br>That you can get rid of me!<br>Ha, that I'm not laughing!<br>You'll never get rid of me!<br>I won't disappear until you never wake up again.
Commenting	P1: Das war aber ein lautes Geräusch.<br>So ein unangenehmes, schrilles Geräusch vom Wecker.<br>Vielleicht wäre ein anderer Klingelton besser.<br>Nun aber schnell anziehen und Zähne putzen.<br>Diesmal musst du pünktlich sein.<br>Du warst letztes Mal ja schon zu spät.<br>Für Duschen reicht nicht mehr die Zeit.<br>Keine frischen Klamotten? Dann ziehst du noch mal die Gestrigen an.<br>Schnell alles einpacken, Türe verschließen.<br>Heute musst du das Auto nehmen.<br>Sonst bist du zu spät.<br>Weißt du noch, wo du geparkt hast?<br>Wahrscheinlich in der Parallelstraße.	P1: But that was a loud noise.<br>Such an unpleasant, shrill noise from the alarm clock.<br>Maybe a different ringtone would be better.<br>Now get dressed and brush your teeth.<br>You have to be on time this time.<br>You were late last time.<br>There is no longer enough time for a shower.<br>No fresh clothes? Then you put on yesterday's clothes again.<br>Pack everything up, lock the door.<br>Today you have to take the car.<br>Otherwise you are too late.<br>Do you remember where you parked?<br>Probably in the parallel street.<br>You couldn't find a parking space for so long.<br>There it is. Get in and buckle up quickly.

Du konntest doch so lange keinen Parkplatz finden.
Da ist es ja. Schnell einsteigen und anschnallen.
Oh, was ist denn da auf der Frontscheibe?
Ist das ein Strafzettel?
Nein, wie ärgerlich.
Man darf hier nicht parken.
Da ist doch ein Schild.
Du hast es übersehen.
Dieses blöde Ordnungsamt.
Das wird dich wahrscheinlich 10 Euro kosten.
Nächstes Mal musst du besser schauen.

Oh, what's that on the windshield?
Is that a ticket?
No, how annoying.
You are not allowed to park here.
There's a sign.
you missed it
This stupid regulatory agency.
This will probably cost you 10 euros.
You'll have to look better next time.
